# Regional and temporal dynamics of DNA methylation and epigenetic gene regulation in response to binge-like alcohol exposure in the adolescent mouse brain

**DOI:** 10.3389/fnmol.2025.1716792

**Published:** 2026-01-05

**Authors:** Amine Cherif, Amine Bourzam, Zeineb Fridhi, Hanani Boukhawiye, Clement Guillou, Pascal Cosette, Sami Zekri, Jérôme Leprince, David Vaudry, Olfa Masmoudi-Kouki

**Affiliations:** 1 Laboratory of Neurophysiology, Cellular Physiopathology and Biomolecules Valorisation, Faculty of Sciences of Tunis, University of Tunis El Manar, Tunis, Tunisia; 2 Laboratory of Neuroendocrine, Endocrine and Germinal Differentiation and Communication (NorDiC), Inserm UMR 1239, University Rouen Normandie, Rouen, France; 3 Univ Rouen Normandie, Inserm US 51, CNRS UAR 2026, HeRacLeS PISSARO, Rouen, France; 4 Confocal Microscopy Unit, Faculty of Medicine of Tunis, University Tunis El Manar, Tunis, Tunisia; 5 Univ Rouen Normandie, Inserm U1245, Normandie Univ, Rouen, France

**Keywords:** binge-like alcohol exposure, adolescent, epigenetic, methylation, oxidative stress, behavior

## Abstract

Adolescence is a critical late phase of the neurodevelopment, characterized by marked brain plasticity and increased vulnerability to environmental challenges such as alcohol exposure. This study examined the impact of binge-like alcohol exposure in male Swiss Webster mice, focusing on oxidative damage, epigenetic and transcriptional alterations in key brain regions, such as the prefrontal cortex, cerebellum, striatum and hippocampus. Our results demonstrated that acute alcohol exposure during adolescence induces oxidative damage with significant alterations in global DNA methylation and gene expression involved in epigenetic regulation with distinct temporal and anatomical profiles. In the prefrontal cortex binge-like alcohol exposure exhibited persistent upregulation of genes associated with DNA methylation and histone deacetylation, consistent with prolonged transcriptional silencing that may impair executive functions and decision-making. The hippocampus appeared particularly sensitive, exhibiting marked decreases in DNA methylation and gene expression changes associated with an open chromatin state leading potentially linked to cognitive impairments in memory and learning impairments in memory and learning. In the striatum, binge-like alcohol exposure induced active DNA demethylation and transient modulation of histone methyltransferases, reflecting a dynamic compensatory response to alcohol-induced transcriptional repression, with implications for reward processing and impulse control. Similarly the cerebellum displayed a biphasic transcriptional pattern suggesting adaptive or homeostatic mechanisms aimed at maintaining cellular and synaptic balance. Collectively, these findings, accompanied by alterations in behavioral tests, highlight the regional specificity of epigenetic remodeling induced by excessive alcohol exposure during adolescence and offer new insights into the molecular mechanisms underlying increased neurodevelopmental vulnerability during this period.

## 1 Introduction

Binge Drinking (BD), characterized by the rapid intake of large quantities of alcohol over a short period, has emerged as a growing public health concern, particularly among adolescents (Lannoy et al., 2021; World Health Organization, 2024). This pattern of alcohol consumption has significant consequences not only for the physical and mental health of young individuals but also for societal well-being (Mojić and Jeličić, 2019). BD is frequently associated with aggressive behaviors, property damage and increased risk of injuries (Chen and Yoon, 2021; Li et al., 2021). Of particular concern, repeated episodes of BD during adolescence critically disrupt brain development, leading to long-lasting impairments in behavior learning and cognitive functions (Williams et al., 2010; Tschorn et al., 2021).

Adolescence is a critical period of neurodevelopment characterized by profound brain maturation, synaptic remodeling, and increased connectivity across brain networks (Blakemore et al., 2010; Spear, 2013; Zhu et al., 2024). This heightened plasticity confers enhanced learning capacity and adaptability but also renders the adolescent brain particularly vulnerable to environmental insults, including psychoactive substances such as alcohol (Doremus-Fitzwater and Spear, 2016; Spear, 2018). Accumulating evidence highlights the deleterious impact of alcohol on the adolescent brain, with BD being particularly damaging (Barbier et al., 2015; Krieger et al., 2018; Lees et al., 2020a, 2020b). One key mechanism underlying these effects involves epigenetic regulation (Mahnke, 2017). BD has been shown to induce alterations in DNA methylation, a process essential for controlling gene expression and highly sensitive to environmental factors such as ethanol exposure (Al Aboud et al., 2023; Zhang and Gelernter, 2017; Asimes et al., 2017). Such methylation changes have been reported in brain regions critical for cognition and emotion, including the prefrontal cortex, amygdala, and hippocampus (Pandey et al., 2015; Edenberg and McClintick, 2018; Kamal et al., 2020). These alterations may disrupt the expression of genes controlling synaptic plasticity and neurotransmission, thereby increasing vulnerability to long-term impairments in memory, learning and behavioral impairments (Crews et al., 2016; Kyzar et al., 2019). In parallel, histone modifications represent another major layer of epigenetic regulation, shaping chromatin structure and transcriptional activity (Tibben and Rothbart, 2024). Alcohol exposure can rapidly trigger these modifications, leading to both immediate and persistent changes in gene expression (Renthal and Nestler, 2008). Interactions between DNA methylation and histone remodeling further orchestrate the transcriptional response to alcohol-induced oxidative stress and neuronal injury (Finegersh and Homanics, 2014; Aroor et al., 2014). Together, these epigenetic alterations may underlie long-lasting disruptions in neurodevelopmental processes during adolescence, ultimately contributing to the heightened susceptibility of adolescents engaged in BD to long-term cognitive, emotional and behavioral impairments (Crews et al., 2023).

The medium- and long-term consequences of BD are well documented (Squeglia et al., 2015; Spear, 2016; Waszkiewicz et al., 2018). Even a single episode can induce persistent changes in brain volume, particularly in the hippocampus, along with deficits in plasticity, memory and learning thereby increasing addiction risk (Maynard and Leasure, 2013; Maurage et al., 2020; Zeganadin et al., 2024). Few studies have examined the immediate epigenetic responses occurring within hours of alcohol exposure, despite evidence that such rapid changes may underlie early behavioral disturbances (Manzardo et al., 2012; Hasselgård-Rowe et al., 2022). Key regions such as the prefrontal cortex and hippocampus are particularly sensitive to ethanol-induced toxicity due to their ongoing maturation, while the cerebellum and striatum are also vulnerable, reflecting the widespread impact of BD on circuits mediating cognition, motor coordination and reward processing (Casey et al., 2008; Kempermann et al., 2015; Brocato and Wolstenholme, 2021; Spear, 2013). Beyond these epigenetic modifications, accumulating evidence indicates that BD engages multiple neurobiological mechanisms that disrupt brain function. Alcohol exposure during adolescence has been shown to induce oxidative stress, neuroinflammation and mitochondrial dysfunction, all of which compromise neuronal integrity and synaptic remodeling (Crews et al., 2016; Alfonso-Loeches and Guerri, 2011).

In this context, the present study investigates the early epigenetic and molecular consequences of a single episode of binge-like alcohol exposure during adolescence, focusing on oxidative stress, DNA methylation, and associated gene expression changes across key brain regions, and examines how these alterations contribute to rapid behavioral impairments.

## 2 Materials and methods

### 2.1 *In vivo* treatment

#### 2.1.1 Animals

Male Swiss Webster mice aged 30 days were obtained from the National Institute Pasteur. Animals were in groups of four per cage in an accredited animal facility (approval number: FST/LNFP/Pro 162019v3) under controlled environmental conditions: 12-h light/dark cycle, temperature maintained at 21 ± 1 °C, and relative humidity at 70 ± 10%. Animals had ad libitum access to standard chow and tap water and were allowed to acclimate prior to experimentation. All procedures were approved by the Animal Ethics Committee of the National School of Veterinary Medicine, Sidi Thabet (approval number: FST/82-CEEA-ENMV), and were conducted in accordance with national and international guidelines for the care and use of laboratory animals.

#### 2.1.2 Experimental design

**Figure 1:**
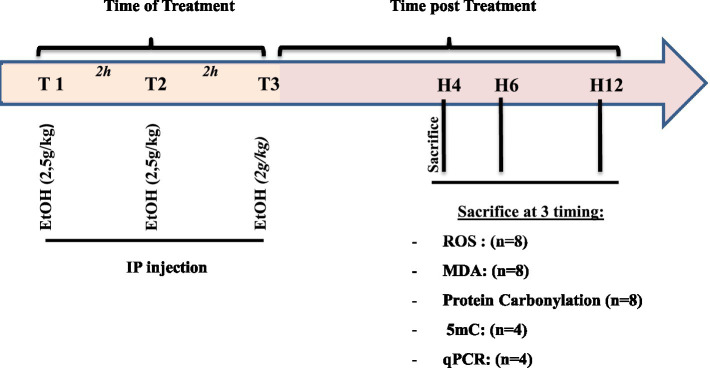
Experimental design of the study showing treatments and assays. Animals received three intraperitoneal injections of ethanol (15%): two doses of 2.5 g/kg followed by 2.0 g/kg (T1: 2.5 g/kg, T2: 2.5 g/kg, T3: 2.0 g/kg) at two-hour intervals. Control animals received equivalent volumes of 0.9% saline. Animals were sacrificed at 4, 6, and 12 h after the final ethanol injection, and brains were collected at these time points for real-time PCR, DNA methylation, and biochemical analyses.

A total of 74 adolescent mice (postnatal day 30, P30) were randomly assigned to two groups: control (*n* = 37) and ethanol-exposed (*n* = 37). The binge-like ethanol exposure protocol consisted of three intraperitoneal injections of ethanol at doses of 2.5 g/kg, 2.5 g/kg, and 2.0 g/kg, administered at 2-h intervals. Ethanol was diluted in 0.9% saline and injected as a 15% (w/v) solution and control animals received equivalent volumes of 0.9% saline (Lacaille et al., 2015; Cherif et al., 2025). Mice were deeply anesthetized and sacrificed at 4, 6, or 12 h following the last alcohol injection of the binge-like alcohol exposure protocol (Figure 1). Brains from ethanol-treated mice and sham controls (*n* = 8 per group per time point) were collected at each designated time point. Each brain was carefully divided into two hemispheres along the median plane. From each hemisphere, the Prefrontal cortex (PFC), striatum (S), hippocampus (H), and cerebellum (Ce) were rapidly dissected and collected for subsequent analyses. Within each subgroup (*n* = 8), four hemispheres were used for quantitative RT-PCR and four hemispheres for global DNA methylation assessment, while the eight corresponding contralateral hemispheres were used for the measurements of oxidative stress markers such as reactive oxygen species (ROS), malondialdehyde (MDA) and protein carbonylation. 96 h following the binge-like alcohol exposure, 13 sham and 13 ethanol-treated mice were subjected to a battery of behavioral tests. The dose of alcohol administered to the mice was determined based on previous results obtained with adolescent and adult mice (Lacaille et al., 2015; Cherif et al., 2025).

### 2.2 Study of epigenetic gene expression

#### 2.2.1 RNA extraction and cDNA synthesis

Animals were lightly anesthetized with 2% isoflurane (Piramal Healthcare, Grangemouth, UK) and sacrificed by decapitation. Four half brains were rapidly dissected, and different distinct brain structures (PFC, S, H, Ce) were isolated and collected in TRIzol reagent (Invitrogen, Carlsbad, CA, USA). Total RNA was extracted using the phenol/chlorophorm method and further purified using a Nucleospin RNA kit (Macherey-Nagel, Hoerd, France). RNA concentration was measured using a NanoDrop ND-2000C spectrophotometer (Thermo Scientific, Wilmington, CA, USA), and RNA quality was evaluated with a Bioanalyzer (Agilent, Massy, France). All samples had an RNA Integrity Number (RIN) greater than 7.5, indicating high-quality RNA. One microgram of total RNA from each sample was converted into single stranded cDNA using the Improm-II reverse transcriptase kit (Promega, Madison, WI, USA) with random primers (0.5 μg/mL).

#### 2.2.2 Real-time PCR

A panel of 11 primer pairs was designed with the Primer express software (Life Technologies, Saint-Aubin, France) and validated for efficiency and specificity (Table 1). cDNA was then amplified in the presence of 1X SYBR Green Mastermix (Applied Biosystems, Courtaboeuf, France) containing preset concentrations of dNTPs and MgCl_2_ with forward and reverse primers using a QuantStudio 12 K Flex (Life Technologies). The relative amount of cDNA in each sample was calculated using the comparative quantification cycle method and expressed as 2^−ΔΔCt^ using *Gapdh*, *Hsp90ab1,* and *Actb* which were constant in all treatment conditions, as internal standards for variation in amounts of input mRNA.

**Table 1:** List of genes included in the panel.

Gene symbol	GenBank accession number	Forward primer	Reverse primer
Dnmt1	NM_001199431.2	AGCCGCTCAAAGCAAAAGTG	TGGGGTTCATCCACAGCATC
Dnmt3a	NM_007872.5	TCAATGTCACCCTGGAGCAC	CTGCAGCAGTTGTTGTTCCC
Dnmt3b	NM_001417019.1	GATGAGGAGAGCCGAGAACG	CAGAGCCCACCCTCAAAGAG
TET1	NM_001253857	ATC ATT CCA GAC CGC AAG AC	AAT CCA TGC AAC AGG TGA CA
TET2	NM_001040400	CCA CAG AGA CCA GCA GAA CA	TCC GCT TTC TTC TTG CAA CT
Ezh2	NM_007971	CAAATCTGTTCAGAGGGAGCAAA	CACTTACGATGTAGGAAGCAGTCATACT
Ehmt1	NM_172545	GGAGCAGCTCGGGTTCG	CACTGGCTTGCCTCTGGG
KAT7	NM_001195003	GGTACTGCTCCGATACCTGC	TCTGAAGGGCTTGCAGAGTG
Cbp	NM_001025432.1	GCGAGCCAGCGAGGA	CGGGCAGGGGATGAG
Hdac1	NM_008228.3	GAGTTCTGTCAGTTGTCCACGG	CTCTTCCACGCCATCGCC
Hdac2	NM_008229.2	CCGGTGTTTGATGGACTCTT	GCGCTAGGCTGGTACATCTC
Actb	NM_007393.5	AGGTCATCACTATTGGCAACGA	CACAGGATTCCATACCCAAGAAG
Gapdh	NM_001411840	CATGGCCTTCCGTGTTCCTA	CCTGCTTCACCACCTTCTTGA
Hsp90ab1	NM_008302.3	CAGAAATTGCCCAGCTCATGT	CCGTCAGGCTCTATATCGAA

### 2.3 Quantification of global DNA methylation (5-methylcytosine)

Fourhalf brain were used to assess global DNA methylation levels. Genomic DNA was extracted using the CTAB as described by Borges et al. (2009). DNA was then purified and hydrolyzed following the protocol established by Gao et al. (2019). The global methylation status, specifically the 5-methylcytosine (5-mC) content, was quantified using the Methylated DNA Quantification Kit (ab117128; Abcam, USA) based on an enzyme-linked immunosorbent assay (ELISA) method. For the quantification of 5-mC. All procedures were carried out according to the manufacturer’s instructions. Absorbance was measured at 450 nm using an automated microplate reader (Agilent BioTek Synergy LX Multimode Reader).

### 2.4 Ingenuity pathway analysis (IPA)

Ingenuity Pathway Analysis (IPA) was used to explore functional interactions among genes, proteins and other molecules, enabling the construction of interaction networks based on curated annotations. Identified genes were classified according to their associated molecular functions and biological processes. Only networks with a statistical score ≥ 40, indicating strong biological relevance, were retained for further analysis.

### 2.5 Measurements of oxidative stress markers

#### 2.5.1 Tissue preparation

Tissues from different brain regions were carefully dissected and homogenized in a lysis buffer containing 1% Triton X-100, 50 mM Tris–HCl, 10 mM EDTA, and protease inhibitors (Pierce EDTA-free, Thermo Scientific, Paris, France). The homogenates were then centrifuged at 14,000 g for 15 min at 4 °C. Protein concentration in each sample was determined using the BCA Protein Assay Kit (Biomaghreb, Tunisia), following the manufacturer’s instructions. Aliquots of the homogenates were stored at −80 °C until oxidative stress marker quantification.

#### 2.5.2 ROS measurement

The levels of reactive oxygen species (ROS) were assessed using the dichlorofluorescein diacetate (DCFH₂-DA) fluorescent probe. An aliquot of 100 μL of brain tissue extract was exposed to DCFH₂-DA (10^−6^ M) for 30 min in the dark at 37 °C. Subsequently, the probe was removed, and the samples were washed twice with PBS to eliminate excess dye. Fluorescence was then recorded at an excitation wavelength of 485 nm and an emission wavelength of 535 nm, using a Bio-Tek FL800TBI microplate reader.

#### 2.5.3 Lipid peroxidation assay: measurement of malondialdehyde

Lipid peroxidation in different structure was assessed using the TBARS (Thiobarbituric Acid Reactive Substances) assay (Draper et al., 1990). MDA, a final product of lipid peroxidation, reacts with thiobarbituric acid (TBA) to form a colored complex, measured at 530 nm. Tissue homogenates were treated with a BHT-TCA solution, centrifuged, and then mixed with HCl and TBA-Tris before being heated at 80 °C. The absorbance of the MDA-TBA complex was determined using UV–visible spectrophotometry. MDA concentrations were calculated using an extinction coefficient of 1.56 × 10^5^ mol/L•cm and expressed as nanomoles of MDA per milligram of protein.

#### 2.5.4 Protein carbonylation assay

Protein carbonylation was quantified using the 2,4-dinitrophenylhydrazine (DNPH) method (Levine et al., 1990). Carbonyl groups react with DNPH to form stable hydrazones, which are detectable by spectrophotometry. Soluble proteins from hippocampal tissues were incubated with a DNPH solution (10 mM in 2.5 M HCl) at 37 °C for 1 h. After precipitation with 30% TCA and centrifugation (11,000 g, 5 min, 4 °C), proteins were washed with an ethyl acetate/ethanol mixture (1,1, v/v) and dissolved in potassium phosphate buffer (20 mM, pH 2.3) containing 6 M guanidine hydrochloride. The absorbance of the hydrazones was measured at 375 nm. The carbonyl concentration was calculated using a molar extinction coefficient of 22,000 M^−1^ cm^−1^ and expressed as nanomoles of carbonyls per milligram of protein.

### 2.6 Behavioral study

To investigate the effects of binge-like alcohol exposure on cognitive abilities in adolescent mice, three behavioral tests targeting relevant brain regions were selected: spontaneous alternation in a T-maze, social memory in the three-chamber test, and anxiety in the elevated plus maze. All assessments were conducted 96 h post-alcohol administration to evaluate lasting cognitive impacts. Behavioral assessments were conducted in a fixed chronological sequence to prevent inter-test interference. Animals were first evaluated for hippocampal-dependent spatial memory using the T-maze spontaneous alternation task, followed by sociability and social memory assessment in the Three-Chamber Test, and finally for anxiety-like behavior in the elevated plus maze (EPM). The same animals were used for all tests, with a minimum 24-h interval between each to allow recovery. All behavioral sessions were video-recorded and analyzed using ANY-maze software (Stoelting Co., USA) and complemented by manual scoring.

#### 2.6.1 Elevated plus maze

The elevated plus maze (EPM) was employed to assess anxiety-like behavior in mice (Pellow et al., 1985). The apparatus, consisting of two open and two closed arms elevated 65 cm above the floor, exploits the conflict between exploratory drive and aversion to open spaces. Each mouse was placed in the central platform facing an open arm for a 5-min trial under ~30 lux illumination. Sessions were video-recorded and scored for time spent and entries in each arm. The maze was cleaned with 70% ethanol between trials. Data were analyzed using paired t-tests with GraphPad Prism 8.

#### 2.6.2 The spontaneous alternation test in a T-maze

The spontaneous alternation T-maze test was used to assess spatial working memory and cognitive flexibility (Delatour and Gisquet-Verrier, 1996; Lalonde, 2002; Deacon and Rawlins, 2006). Mice performed ten trials, each starting from the stem arm and choosing freely between two goal arms. An entry was scored when all four paws crossed, after which the arm was closed for 30 s. Animals were then placed in an opaque box during a 70-s inter-trial interval. Behavioral measures included arm choice, latency, and alternation rate, defined as selecting a different arm than in the previous trial. Alternation performance was analyzed using a one-tailed Student’s t-test (GraphPad Prism 8).

#### 2.6.3 The three-chamber

The Three-Chamber Social Interaction Test assessed sociability and social memory in mice. The apparatus consisted of three interconnected chambers. After a 10-min habituation, the sociability phase was conducted by placing an unfamiliar conspecific (stranger 1) in one side chamber and an empty cage in the other; time spent interacting with each was measured. In the social novelty phase, a novel conspecific (stranger 2) replaced the empty cage, allowing assessment of preference for social novelty. Behavioral parameters included interaction time and the social preference index (SPI, %). Each session lasted 10 min and was video-recorded. The apparatus was cleaned with 70% ethanol between trials. Data were analyzed using a one-sample t-test or Wilcoxon signed-rank test depending on distribution, with significance set at *p* < 0.05 (GraphPad Prism 8).

### 2.7 Statistical analysis

All molecular data were analyzed using GraphPad Prism (version 8, GraphPad Software, San Diego, CA, USA). The results are expressed as mean ± standard error of the mean (SEM). The effects of ethanol exposure and time were evaluated using a two-way analysis of variance (ANOVA), with treatment (EtOH-treated animals *vs*. sham) and time points 4 h, 6 h and 12 h as fixed factors. When a significant main effect or interaction was detected, Tukey’s *post hoc* multiple comparison test was applied to identify specific group differences. Statistical significance was set at *p* < 0.05. The IPA networks are ranked based on a score calculated as the negative logarithm of the *p*-value [−log(*p*-value)] derived from statistical testing. A higher score indicates a lower probability that the observed association occurred by chance, suggesting a stronger biological significance. Additionally, IPA provides a Z-score that reflects the deviation between the observed and expected activation patterns within a pathway. A high Z-score indicates significant biological relevance and potential activation or inhibition of the pathway.

## 3 Results

### 3.1 Adolescent binge-like alcohol exposure induces ROS accumulation and oxidative damage

**Figure 2:**
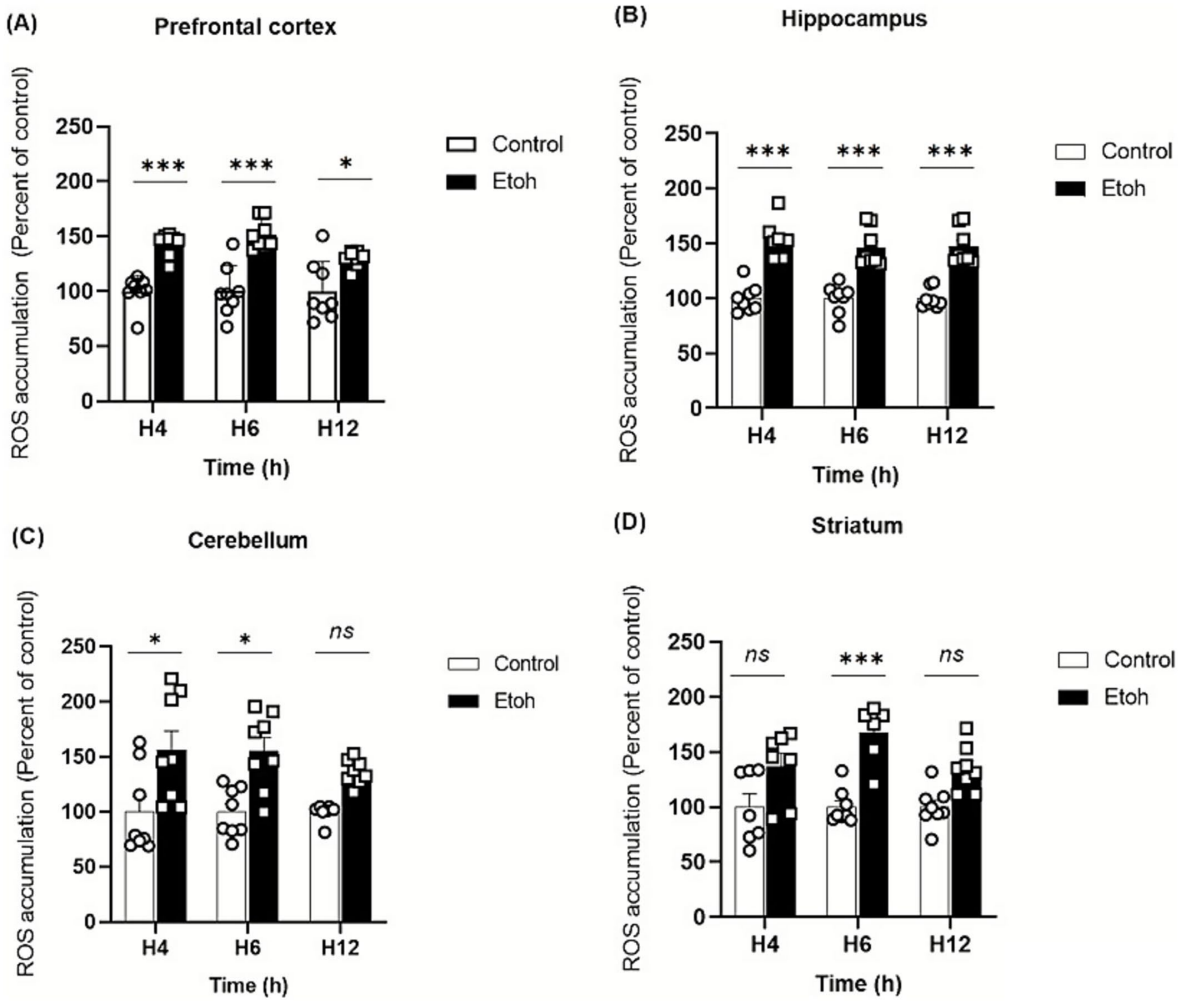
Effect of alcohol consumption on reactive oxygen species accumulation across different brain regions following acute binge ethanol exposure. **(A)** Prefrontal cortex, PFC: ROS levels were significantly increased in the EtOH group compared to control at 4 h, 6 h, and 12 h post-intoxication (Two-way ANOVA: treatment effect *F*(1,42) = 68.43, *p* < 0.0001; *post hoc* Tukey: *p* = 0.0002 at 4 h, *p* < 0.0001 at 6 h, *p* = 0.0215 at 12 h). **(B)** Hippocampus: A strong elevation of ROS levels was observed in the EtOH group at all time points (Two-way ANOVA: F(1,42) = 140.3, *p* < 0.0001; post hoc Tukey: *p* < 0.0001 at 4 h, 6 h, and 12 h). **(C)** Cerebellum: ROS levels were transiently increased at 4 h and 6 h post-intoxication in EtOH-treated mice (Two-way ANOVA: F(1,42) = 21.30, p < 0.0001; post hoc Tukey: *p* = 0.0439 at 4 h and *p* = 0.0486 at 6 h). **(D)** Striatum: ROS levels were significantly reduced at 6 h post-intoxication in the EtOH group compared to control, with no difference at 4 h and 12 h (Two-way ANOVA: *F*(1,36) = 34.66, p < 0.0001; post hoc Tukey: *p* = 0.0004 at 6 h). Each value represents the mean of at least six mice. Data are presented as mean ± SEM. Statistical significance was determined using Tukey’s multiple comparison test (*p < 0.05, **p < 0.01, ***p < 0.001, ns non significant *vs* saline treated mice).

**Figure 3:**
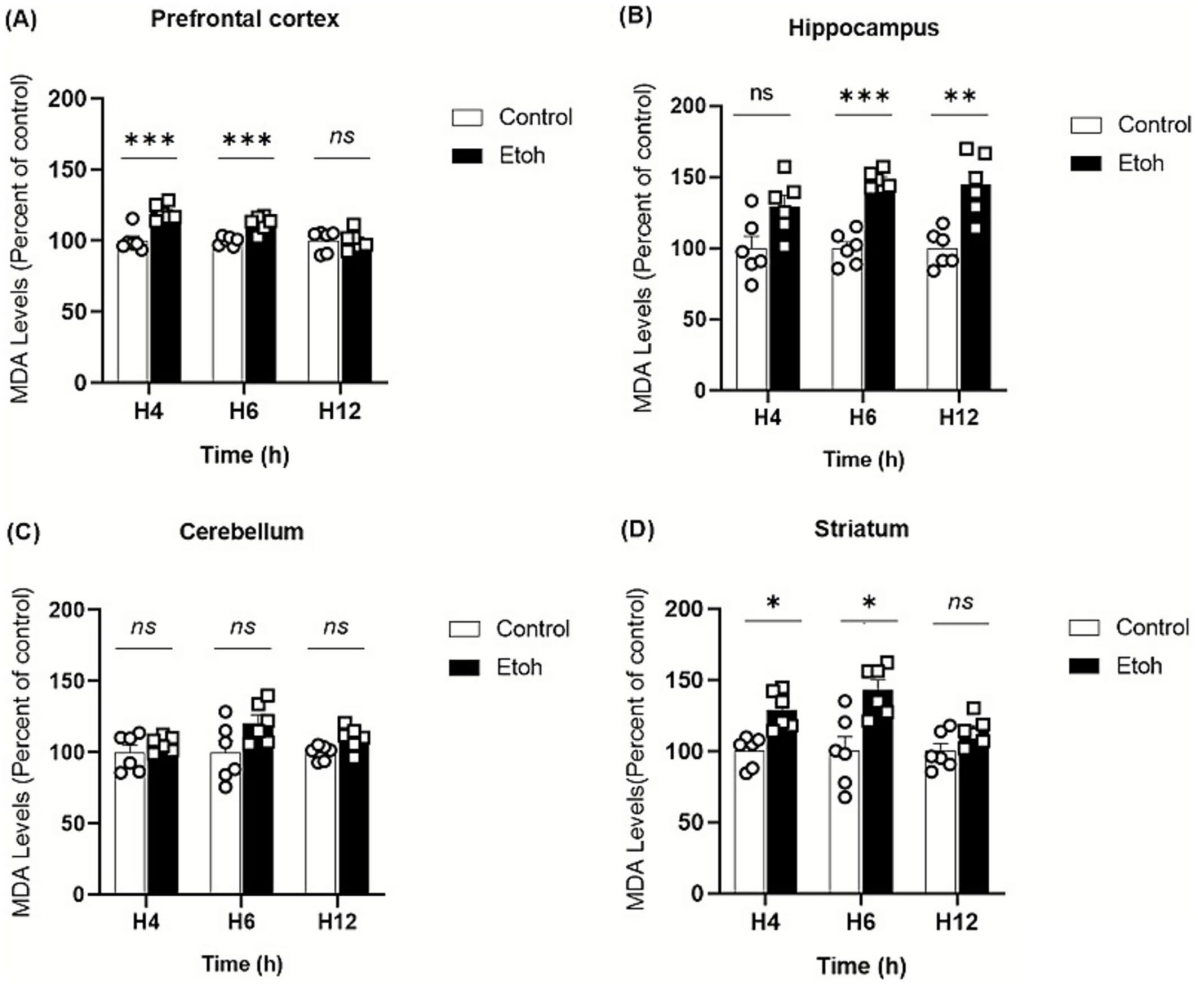
Spatiotemporal variation of MDA content in tissue extracts in response to oxidative stress induced by binge-like alcohol exposure. **(A)** In the prefrontal cortex, lipid peroxidation was significantly increased in the EtOH group compared to controls at 4 h and 6 h post-intoxication (Two-way ANOVA: treatment effect *F*(1,30) = 26.51, *p* < 0.0001; post hoc Tukey: *p* < 0.0001 at 4 h and *p* < 0.0001 at 6 h). No significant difference was observed at 12 h. **(B)** In the hippocampus, a significant treatment effect was detected (Two-way ANOVA: F(1,30) = 47.42, *p* < 0.0001). *Post hoc* Tukey’s test revealed a marked increase in MDA levels in the EtOH group at 6 h (*p* = 0.0008) and 12 h (*p* = 0.0017), while no change was detected at 4 h. **(C)** In the cerebellum, Two-way ANOVA showed a significant treatment effect (F(1,30) = 9.158, *p* = 0.0050), although Tukey’s post hoc test did not reveal any significant differences between groups at the analyzed time points. **(D)** In the striatum, Two-way ANOVA indicated a significant main effect of ethanol treatment on MDA levels (F(1,30) = 29.66, *p* < 0.0001). Tukey’s post hoc analysis showed a significant rise in MDA at 4 h (*p* = 0.0363) and 6 h (*p* = 0.017) in ethanol-treated mice compared to controls. Each value represents the mean of at least six mice. Data are presented as mean ± SEM. Statistical significance was determined using Tukey’s multiple comparison test (*p < 0.05, **p < 0.01, ***p < 0.001, ns non significant *vs* saline treated mice).

**Figure 4:**
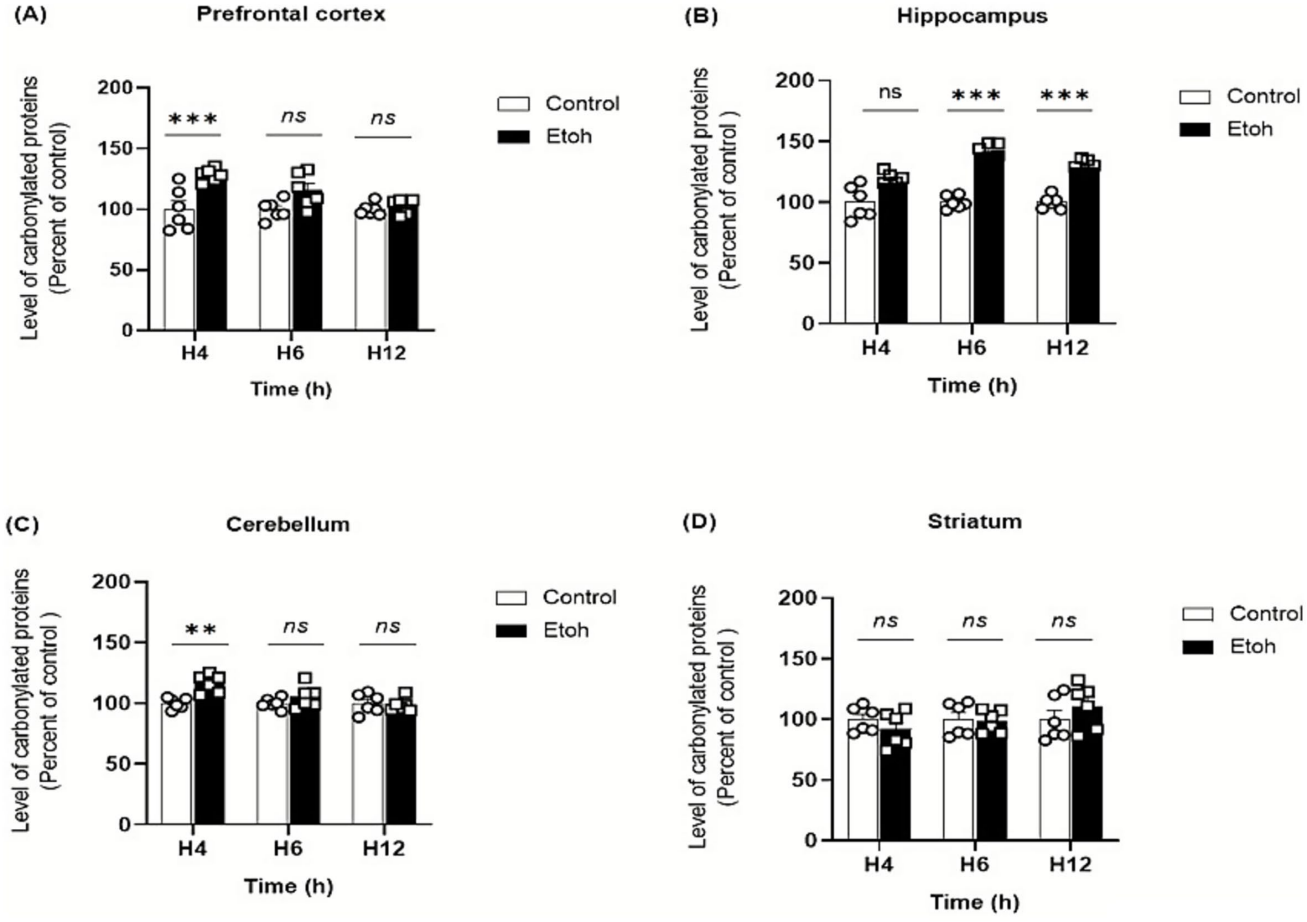
Spatiotemporal quantification of protein carbonyl levels in brain tissue extracts in response to oxidative stress induced by binge-like alcohol exposure. **(A)** Prefrontal cortex: Two-way ANOVA revealed a significant main effect of ethanol treatment (F(1,30) = 21.15, *p* < 0.0001), although Tukey’s post hoc test did not indicate significant differences between groups at the analyzed time points. **(B)** Hippocampus: Two-way ANOVA revealed a significant main effect of ethanol treatment (F(1,30) = 87.05, *p* < 0.0001). Tukey’s post hoc test demonstrated a marked elevation of protein carbonyl levels in ethanol-treated mice compared to controls at 6 h (*p* < 0.0001) and 12 h (*p* < 0.0001). **(C)** Cerebellum: Two-way ANOVA revealed a significant main effect of ethanol treatment (F(1,30) = 8.923, *p* = 0.0056). Tukey’s post hoc test showed a significant increase in protein carbonyl levels at 4 h in ethanol-treated mice compared with controls (*p* = 0.0034). No significant differences were observed at 6 h or 12 h between control and ethanol groups. **(D)** Striatum: Levels of protein carbonyls did not differ significantly across groups. Two-way ANOVA showed no significant main effect or interaction (F(1,30) = 0.003, *p* = 0.9551). Post hoc Tukey’s multiple comparisons confirmed the absence of significant differences between control and ethanol-exposed mice at all time points. Each value represents the mean of at least six mice. Data are presented as mean ± SEM. Statistical significance was determined using Tukey’s multiple comparison test (*p < 0.05, **p < 0.01, ***p < 0.001, ns non significant *vs* saline treated mice).

Ethanol neurotoxicity is closely associated to oxidative stress, therefore, we measured ROS levels in different brain regions using the CM-H₂DCFDA probe, which fluoresces upon oxidation. As shown in Figure 2, binge-like ethanol exposure induced a marked increase in ROS production with distinct temporal and regional profiles. ROS levels increased significantly in the prefrontal cortex (+43%; Figure 2A) and hippocampus (+53%; Figure 2B) as early as 4 h after ethanol exposure and remained significantly elevated up to 12 h (prefrontal cortex: +29%; Figure 2A; hippocampus: +46%; Figure 2B). The cerebellum also showed an early but transient increase in ROS levels (Figure 2C), while the striatum showed a significant elevation at 6 h (+67%: Figure 2D). These results indicate that adolescent brains are particularly vulnerable to acute ethanol-induced oxidative stress. To further confirm oxidative damage, MDA, a product of lipid peroxidation, was quantified. As shown in Figure 3, MDA levels increased significantly in the prefrontal cortex (+19% at 4 h and +12% at 6 h; Figure 3A), hippocampus (+47% at 6 h and +48% at 12 h; Figure 3B) and striatum (+29% at 4 h and +42% at 6 h; Figure 3D). Moreover, as shown in Figure 4, the hippocampus showed a marked and sustained increase in protein carbonyl levels (+40% at 6 h; +28% at 12 h; Figure 4B), whereas the cerebellum showed a transient elevation at 4 h. The hippocampus is distinguished by a strong and sustained production of ROS, with a sustained elevation in MDA and protein carbonyl levels.

### 3.2 Effect of binge-like alcohol exposure on epigenetic modifications in the adolescent brain

To investigate the effects of binge-like alcohol exposure on global DNA methylation in adolescent mice, we quantified 5mC levels in the prefrontal cortex, hippocampus, cerebellum and striatum at 4, 6, and 12 h after administration of ethanol following a binge-like protocol (2.5 g/kg, 2.5 g/kg, and 2 g/kg at 2-h intervals).

**Figure 5:**
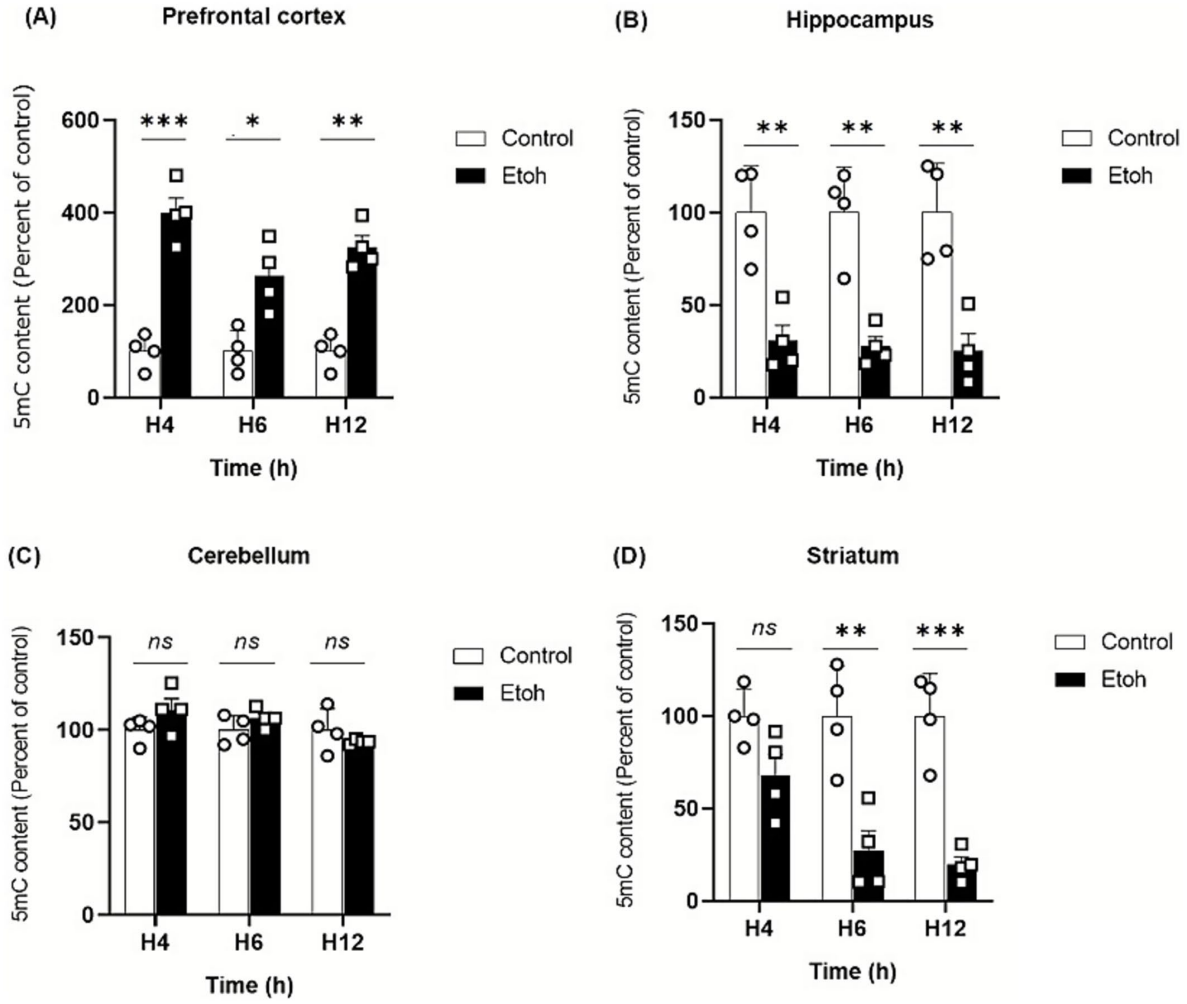
Evolution of global DNA methylation by evaluating 5-methylcytosine (5mC) in adolescent mice. **(A)** Prefrontal cortex: Two-way ANOVA revealed a significant main effect of treatment (*F*(1,18) = 16.75, *p* < 0.0001). Post hoc Tukey’s test confirmed a persistent increase in 5-mC levels in ethanol-exposed mice compared to controls at 4 h (*p* < 0.0001), 6 h (*p* < 0.0001) and 12 h (*p* = 0.0009), indicating sustained hypermethylation in the PFC. **(B)** Hippocampus: A significant main effect of treatment was observed (F(1,18) = 70.54, *p* < 0.0001). Post hoc Tukey’s comparisons revealed a marked decrease in 5-mC levels in ethanol-treated groups relative to controls at 4 h (*p* = 0.0023), 6 h (*p* = 0.0015) and 12 h (*p* = 0.0011), indicating pronounced hypomethylation in the hippocampus. **(C)** Cerebellum: No significant effect of treatment or interaction was found (F(1,18) = 1.23, *p* = 0.28), indicating no detectable change in global 5-mC following acute ethanol exposure. **(D)** Striatum: Two-way ANOVA demonstrated a strong treatment effect (F(1,18) = 54.21, *p* < 0.0001). Post hoc Tukey’s test showed a significant reduction in 5-mC levels in the ethanol group at 6 h (*p* = 0.0011) and 12 h (*p* = 0.0004) compared to controls, suggesting time-dependent DNA demethylation. Each value represents the mean of at least four mice. Data are presented as mean ± SEM. Statistical significance was determined using Tukey’s multiple comparison test (*p < 0.05, **p < 0.01, ***p < 0.001, ns non significant *vs* saline treated mice).

Our findings indicate region-specific effects of binge-like alcohol exposure on DNA methylation across these brain regions. In the prefrontal cortex, alcohol exposure induced a significant increase in global DNA methylation as early as 4 h which persisted up to 12 h (Figure 5A). In contrast, no significant changes were detected in the cerebellum over the same treatment period (Figure 5C). Meanwhile, both the hippocampus and striatum exhibited a reduction in global DNA methylation (Figures 5B,C). In the hippocampus, this decrease was observed as early as 4 h and remained significant up to 12 h. In the striatum, the decline appeared slightly later, becoming detectable at 6 h and persisting until 12 h.

### 3.3 Effect of binge-like alcohol exposure on the expression of genes involved in epigenetics mechanisms

**Figure 6:**
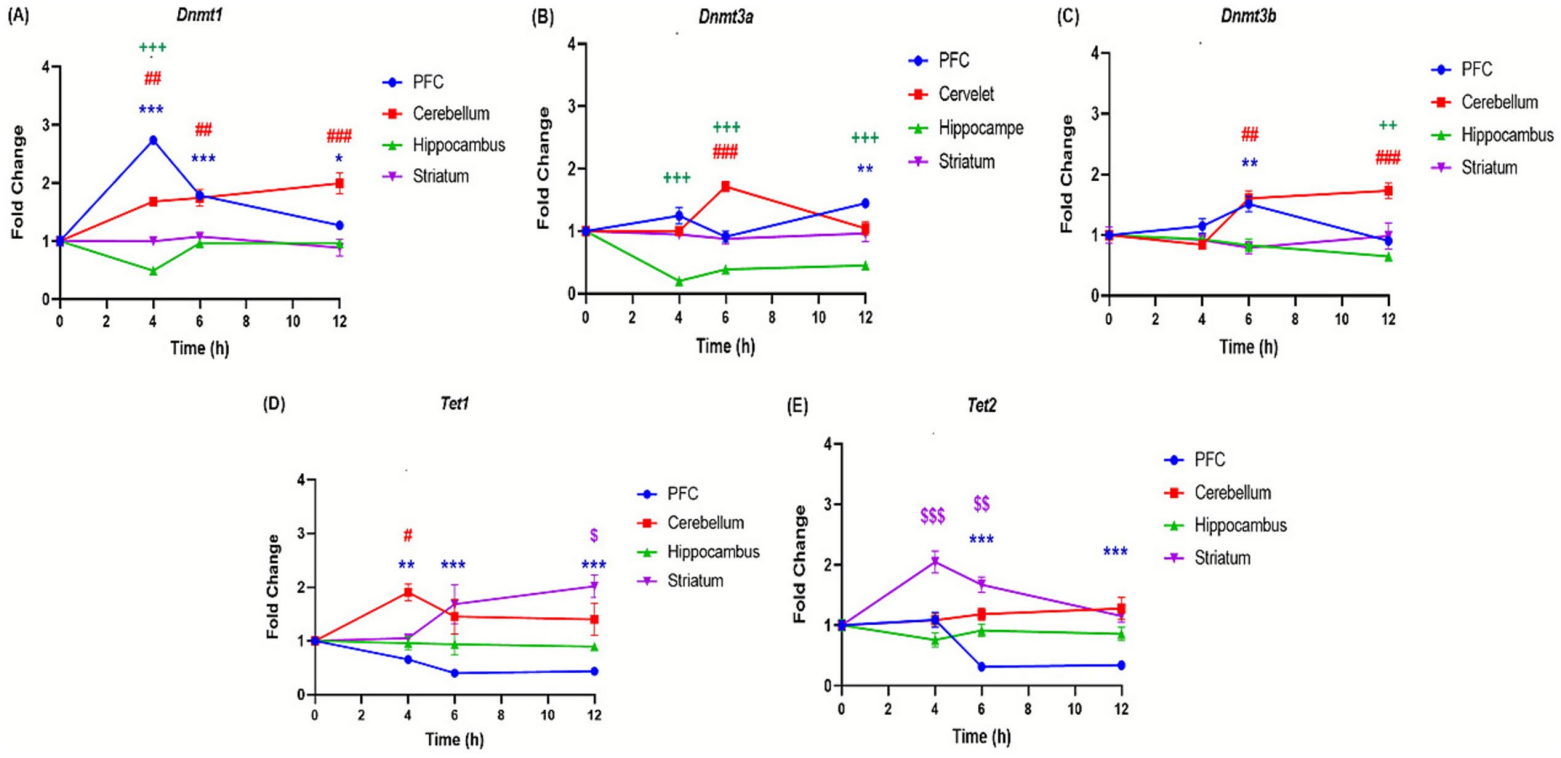
Effect of ethanol injections on the expression of genes involved in DNA methylation in the brain of adolescent mice exposed to a BD. Quantification of mRNA levels of Dnmt1 **(A)**, Dnmt3a **(B)**, Dnmt3b **(C)**, Tet1 **(D)**, Tet2 **(E)**, in different brain structures PFC (bleu curves), cerebellum (red curves), hippocampus (green curves), and striatum (purple curves), Time-course effect of ethanol administration on gene expression in different brain structures over after 4, 6 and 12 h after a single binge-like episode of ethanol (2.5 g/kg twice plus 2 g/kg 2-h intervals). Each value represents the mean (± SEM) from four animals. $, #, +, *p < 0.05; $$, ##, ++, **p < 0.01; $$$, ###, +++, ***p < 0.001 versus control. Each data point represents an independent group of animals at the indicated time point; lines connecting points are for visual comparison only and do not indicate repeated measures within the same subjects.

**Figure 7:**
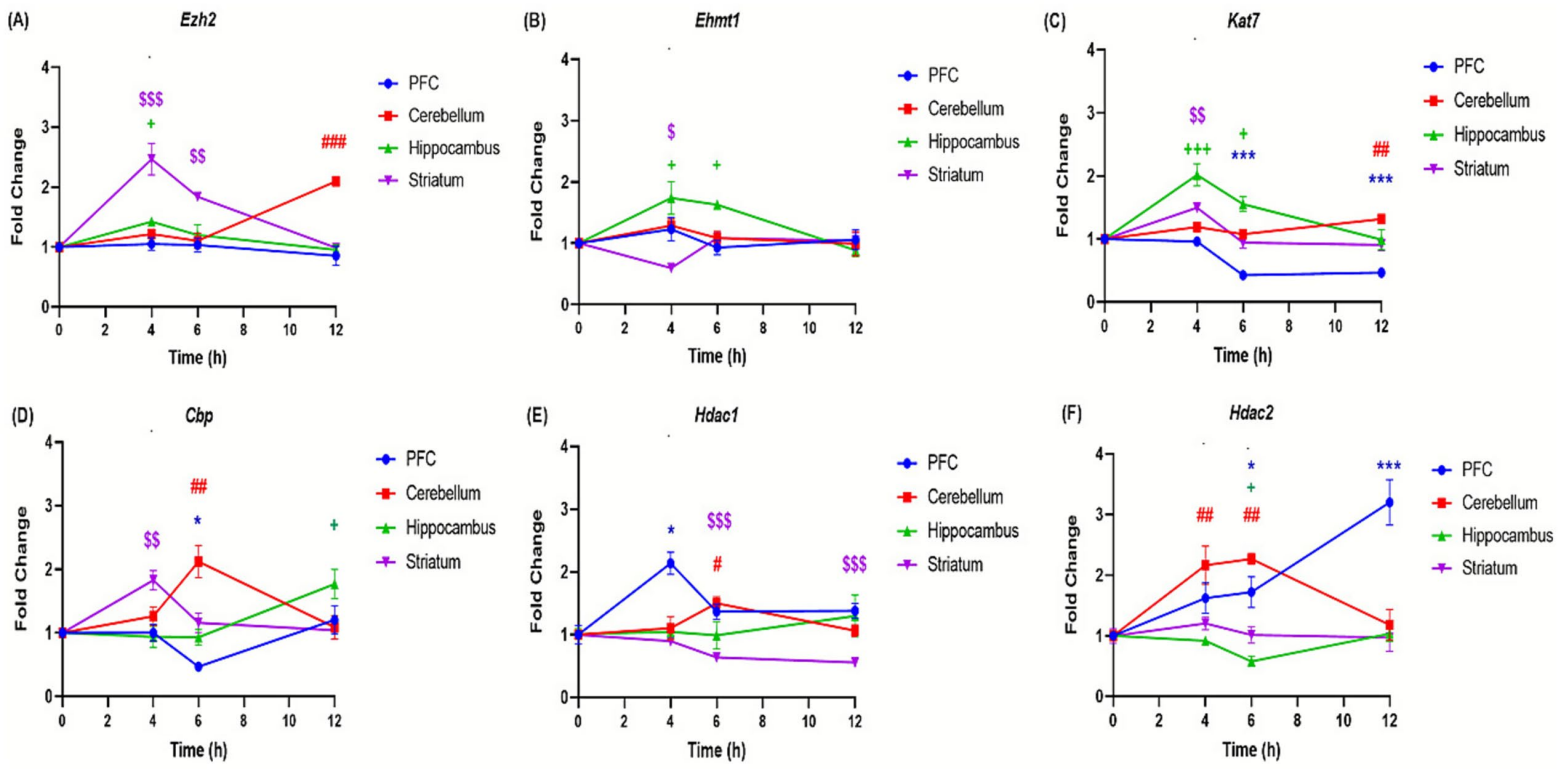
Effect of ethanol injections on the expression of genes involved in histone modifications in the brain of adolescent mice exposed to a binge-like alcohol exposure. Quantification of mRNA levels of Ezh2 **(A)**, Ehmt1 **(B)**, Kat7 **(C)**, Cbp **(D)**, Hdac1 **(E)**, and HDAC2 **(F)**, in different brain structures PFC (bleu curves), cerebellum (red curves), hippocampus (green curves), and striatum (purple curves), **(A,F)** Time-course effect of ethanol administration on gene expression in different brain structures over after 4, 6 and 12 h after a single binge-like episode of ethanol (2.5 g/kg twice plus 2 g/kg 2-h intervals). Each value represents the mean (± SEM) from four animals. $, #, +, *p < 0.05; $$, ##, ++, **p < 0.01; $$$, ###, +++, ***p < 0.001 versus control. Each data point represents an independent group of animals at the indicated time point; lines connecting points are for visual comparison only and do not indicate repeated measures within the same subjects.

The effects of binge-like alcohol exposure on adolescent mice were examined by analyzing the expression of 11 genes implicated in epigenetic regulation (Table 1). The analysis revealed distinct temporal and regional patterns. In the prefrontal cortex, DNA methyltransferase genes, i.e., *Dnmt*1, *Dnmt3a*, *Dnmt3b* (Figures 6A–C) and histone deacetylases *Hdac1 and Hdac2* genes (Figures 7D,E) were upregulated at specific time points such as *Dnmt1* (+173% at 4 h); *Hdac1* (+113% at 4 h); *Dnmt3a* (+44% at 12 h) and *Hdac2* (+220% at 12 h). However, *Tet1*, *Tet2*, *Kat*, and Cbp genes are significantly downregulated, i.e., *Tet1* (−56% at 12 h; Figure 6D), *Tet2* (−66% at 12 h; Figure 6E), *Kat7* (−54% at 12 h; Figure 7C), and Cbp (−53% at 6 h; Figure 7D). In contrast, the cerebellum exhibited sustained upregulation across most time points. Notable and significant increases were observed in DNA methyltransferases genes *Dnmt* (+99% at 12 h) and *Dnmt3a*: (+71% at 6 h) (Figures 6A,B), histone deacetylases genes *Hdac1* (+50% at 6 h) and *Hdac2* (+126% at 6 h) (Figures 7E,F) and acetyltransferases genes *Cbp* (+112% at 6 h) and *Kat7* (+31% at 12 h) (Figures 7C,D). The hippocampus displayed a distinct profile, characterized by downregulation of DNA methyltransferases genes *Dnmt1, Dnmt3a, and Dnmt3b* (D*nmt1*: −51% at 4 h; *Dnmt3a*: −71% at 4 h; *Dnmt3b*: −35% at 12 h; Figures 6A–C) and reduced expression of the histone deacetylase gene *Hdac* (−43% at 6 h) (Figure 7F). Conversely, histone methyltransferases (*Ezh2, Ehmt1*) and acetyltransferases (*Kat7, Cbp*) were upregulated, particularly at 4 h: (*Ezh2:* + 42%; Ehmt1: +73%; *Kat7:* 100%; Figures 7A–C) and 6 h: (*Ehmt1:* +61%; *Kat7:* +55%; Figures 7B,C). The striatum was characterized by persistent upregulation of *Tet1* and *Tet2* gene with a maximum of expression observed at 6 h (*Tet1*: +68%; *Tet2*: +67%; Figures 6D,E), early induction of *Kat7* gene (+49%; Figure 7C) and *Cbp* gene (+82%; Figure 7D), and transient increases in *Ezh2* gene +146% at 4 h (Figure 7A) and repression of Hdac1 gene at 6 h − 37% (Figure 7E).

**Figure 8:**
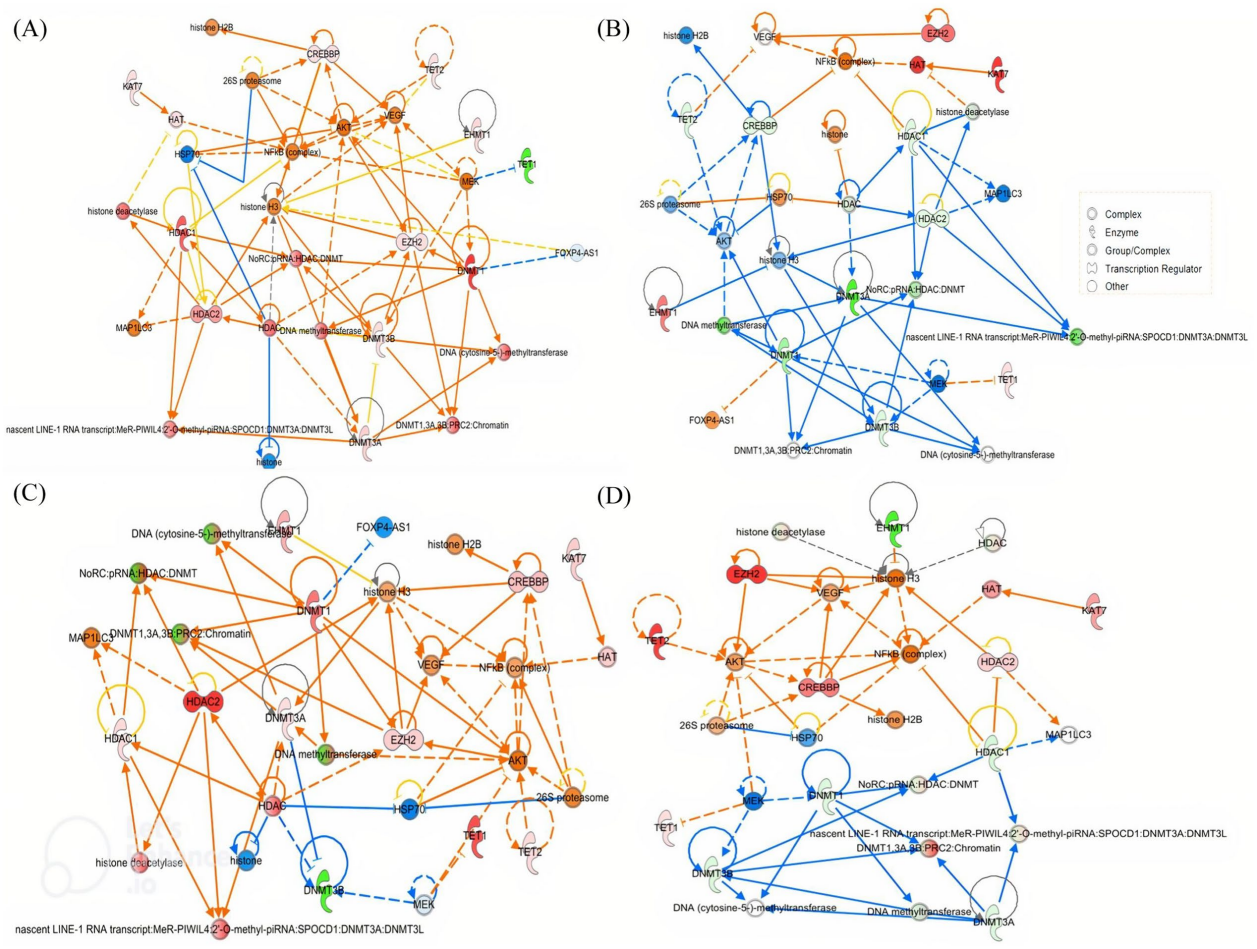
Networks of commonly regulated genes in the brain of adolescent mice exposed to binge-like alcohol exposure. PFC **(A)**, hippocampus **(B)**, cerebellum **(C)**, and striatum **(D)** after 4 h of BD. The regulatory interactions, node colors, and shapes are also explained. The network, named Cellular Assembly and Organization, DNA Replication, Recombination and Repair, and Gene Expression, achieved a relevance score of 40. Nodes represent molecules, and their shape indicates molecular type according to IPA conventions (circle = complex, oval = enzyme, semicircle = transcription regulator, other shapes = additional functional classes). Node colors reflect differential expression: red = upregulated, green = downregulated, orange/yellow = intermediate or moderate change, blue = downregulation in another comparison/group. Edges represent predicted regulatory interactions curated in the Ingenuity Knowledge Base: solid arrows indicate direct interactions, dashed arrows indicate indirect interactions; arrowheads indicate activation, blunt ends indicate inhibition. Connector nodes not measured in the dataset are shown in neutral colors (white/light gray). The network encompasses key regulators of DNA methylation, histone modification, and chromatin remodeling, as well as downstream signaling pathways. A key for node shapes is provided in the inset panel.

Ingenuity Pathway Analysis (IPA) of the 11 differentially expressed genes revealed a significantly regulated network associated with cellular assembly and organization Figure 8. Acute alcohol exposure acted as a potent epigenetic stimulus, inducing a coordinated rearrangement of chromatin states. The response was predominantly characterized by predicted activation of repressive mechanisms (DNMTs, HDACs, PRC2), suggesting a rapid adaptation aimed at restricting the expression of plasticity-related genes, particularly in the prefrontal cortex and hippocampus. This profile was accompanied by enrichment of key regulators, including REST and MECP2, along with predicted activation of NF-κB and CREB/p300 signaling pathways. The involvement of pro-inflammatory mediators and intracellular signaling components further indicated a role for inflammatory and transcriptional regulation in modulating adaptive plasticity processes linked to the reinforcing effects of alcohol.

### 3.4 Adolescent binge-like alcohol exposure induces behavior deficits

**Figure 9:**
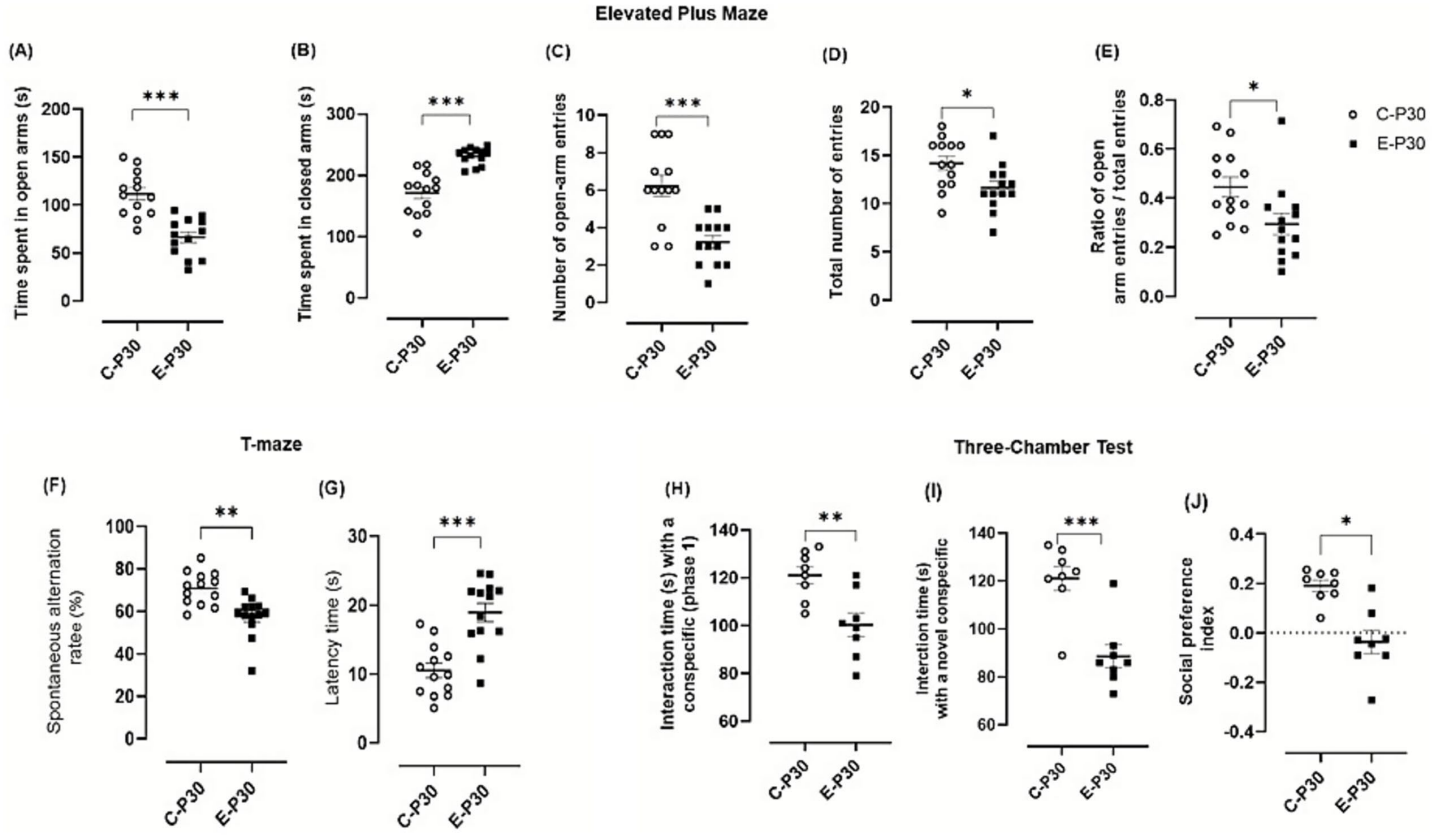
Behavioral assessment. **(A-E)** Effect of binge-like alcohol exposure on anxiety-like behavior in the Elevated Plus Maze: **(A)** time spent in open arms, **(B)** time spent in closed arms, **(C)** number of open-arm entries, **(D)** total number of entries, and **(E)** ratio of open-arm entries to total entries. Data represent the mean ± SEM of 13 mice. After verification of normality (Shapiro–Wilk test), group comparisons were performed using one-way ANOVA followed, when significant, by Tukey’s post hoc test. **(F–G)** Spontaneous alternation test in a T-maze: **(F)** percentage of spontaneous alternations and **(G)** latency to decision. Data represent the mean ± SEM of 13 mice. Spontaneous alternation rate was analyzed using a one-tailed Student’s t-test, followed, when appropriate, by Tukey’s post hoc test. **(H, J)** Three-Chamber Social Interaction Test: evaluation of social memory in a three-compartment paradigm. **(H)** Times spent interacting with a conspecific, **(I)** time spent interacting with a novel conspecific (mouse 2), and **(J)** social preference index (SPI, %). Data represent the mean ± SEM of 8 mice. Paired Student’s t-tests were applied when normality conditions were met (Shapiro–Wilk test). Comparisons: Ethanol-treated P30 vs. control P30. ns = not significant; **p < 0.01; ***p < 0.001.

Adolescent mice were subjected to behavioral tests to evaluate anxiety-like behavior, exploratory activity, spatial memory and social behavior Figure 9.

In the EPM test, ethanol-treated mice displayed a marked anxiety-like phenotype. Animals spent significantly less time in the open arms compared to control (control animals: 111.57 ± 6.46 s; Ethanol-treated animals: 66.39 ± 5.54 s; difference: 45.18 ± 8.51, *p* < 0.0001; Figure 9A) and more time in the closed arms (Control: 171.48 ± 9.45 s; Ethanol: 231.50 ± 3.88 s; difference: 60.02 ± 10.23, *p* < 0.0001; Figure 9B). The number of open arm entries (control animals 6.23 ± 0.56; Ethanol-treated animals: 3.23 ± 0.34; difference: 3.00 ± 0.66, *p* = 0.0001; Figure 9C. C) and the total number of entries (control animals: 14.15 ± 0.73; Ethanol-treated animals: 11.61 ± 0.67; difference: 2.53 ± 0.99, *p* = 0.017; Figure 9D) were both significantly reduced in the ethanol group.

Exploratory activity was also markedly reduced in ethanol-exposed mice, as indicated by significant decreases in locomotor activity and exploratory behaviors, (Figures 9E,F).

In the spontaneous alternation test, ethanol-treated mice showed significant impairments in spatial working memory with reduced spontaneous alternation (control animals: 170.80 ± 2.14; Ethanol-treated animals 157.56 ± 2.59; difference: −13.25 ± 3.36, *p* = 0.006; Figure 9F) and increased latency to first arm entry (control animals: 10.53 ± 1.04 s; Ethanol-treated animals: 18.95 ± 1.34 s; difference: 8.42 ± 1.70, *p* < 0.0001; Figure 9H), indicating slower exploration and impaired memory behavior.

In the three-chamber social interaction test, ethanol-exposed mice exhibited clear deficits in social behavior. During the sociability phase, time spent exploring the familiar conspecific was significantly reduced (control animals: 121.00 ± 3.58 s; Ethanol-treated animals: 100.25 ± 4.95 s; difference: −20.57 ± 7.00, *p* = 0.044; Figure 9H). During the social novelty phase, exploration of the novel conspecific was also decreased (control animals: 121.12 ± 5.06 s; Ethanol-treated animals: 88.63 ± 8.82 s; difference: −32.50 ± 7.00, *p* = 0.0004), resulting in a lower social preference index (Figure 9I). Together, these findings indicate reduced social motivation and recognition following adolescent binge-like alcohol exposure exposure.

## 4 Discussion

In this study, we examined the impact of binge-like alcohol exposure on oxidative stress, epigenetic regulation and behavioral in adolescent mice. In a previous study using the same binge-like ethanol exposure protocol, we showed that BACs increase and remain above 150 mg/dL for up to 7 h after the last ethanol administration (Cherif et al., 2025), indicating that the animals are exposed to the toxic effects of ethanol. Here, we show that binge-like ethanol exposure induces oxidative stress, alters DNA methylation and disrupts the expression of key epigenetic regulators leading to behavioral deficits. These results highlight epigenetic mechanisms as central mediators of ethanol-induced neurotoxicity and underscore adolescence as a critical window of vulnerability.

Oxidative stress is a key mechanism underlying ethanol-induced neurotoxicity and ROS playing a central role in neuronal injury and dysfunction (Albano, 2006; Haorah et al., 2008; Zakhari, 2013; Prakash et al., 2022). In agreement with previous studies, our data demonstrate that binge-like ethanol exposure markedly increased ROS levels in several brain regions, particularly in the frontal cortex, hippocampus, and cerebellum. This excessive ROS production was accompanied by the accumulation of lipid peroxidation products, further confirming oxidative damage (Albano, 2006; Das and Vasudevan, 2007). Importantly, adolescent mice exhibited an earlier and more robust increase in ROS compared to adults, indicating an age-dependent vulnerability of the developing brain to ethanol toxicity (Crews and Nixon, 2009; Coleman et al., 2014). This heightened susceptibility during adolescence may be explained by the immaturity of endogenous antioxidant defense systems and the increased metabolic demand associated with brain development (Guerri and Pascual, 2019; Montesinos et al., 2016; Prakash et al., 2022).

Our results demonstrate that binge-like alcohol exposure induces region-specific epigenetic alterations in the adolescent brain, consistent with previous reports indicating that binge-like alcohol exposure during adolescence induces specific epigenetic alterations in the brain, particularly affecting regions such as the prefrontal cortex, amygdala, and hypothalamus (Kyzar et al., 2019; Brocato and Wolstenholme, 2021; Heidari et al., 2024). Indeed, Wolstenholme et al. (2017) reported alterations in myelin-related gene expression as well as changes in chromatin remodeling genes, suggesting a lasting epigenetic reorganization associated with cognitive deficits and increased alcohol sensitivity. McClintick et al. (2018) demonstrated a large-scale modulation of gene expression in the ventral hippocampus, with 416 genes altered. Similarly, in the nucleus accumbens, hypomethylation of OPRL1 has been linked to heightened reward sensitivity and increased alcohol intake McClintick et al. (2018). Conversely, some structures such as the amygdala exhibit a distinct dynamic, characterized by an early decrease in *DNMT* activity followed in adulthood by hypermethylation of genes such as Neurpeptide Y (Npy) and Brain Derived-Neurotorphic Factor (Bdnf) exon IV. within the prefrontal cortex findings are divergent depending on the specific locus examined. Some studies report hypomethylation of promoters of genes such as Bdnf and Fos-B, leading to gene upregulation (Roth et al., 2009), whereas others describe hypermethylation and histone modifications in synaptic plasticity–related genes, including c-Fos, Cdk5, and Fos-B (Wolstenholme et al., 2017; McClintick et al., 2018; Brocato and Wolstenholme, 2021). Overall, these results indicate that adolescent binge-like alcohol exposure induces heterogeneous epigenetic modifications, which vary according to brain region and targeted genes, rather than following a uniform pattern of hyper- or hypomethylation.

Among the brain regions analyzed, the prefrontal cortex emerged as particularly vulnerable to binge-like alcohol exposure. We observed a pronounced increase in DNA methylation and in the expression of transcriptional repressors, including DNA methyltransferases genes *Dnmt1, Dnmt3a/b* and histone deacetylases (*Hdac1/2*) genes, both of which repress gene expression and synaptic plasticity, processes that are fundamental for cognition (Klose and Bird, 2006; Renthal and Nestler, 2009). Importantly, pharmacological studies show that inhibition of DNMTs and HDACs reduces alcohol intake in animal models (Palmisano and Pandey, 2017; Cano-Cebrián et al., 2021), raising the possibility that these enzymes may serve as therapeutic targets in alcohol use disorders (Abel and Zukin, 2008; Jeanblanc et al., 2015; Han et al., 2023). Conversely, enzymes that facilitate gene transcription and chromatin accessibility, such as *Tet1/2, Kat7*, and *Cbp*, were downregulated following binge-like alcohol exposure, consistent with previous reports linking alcohol consumption to chromatin condensation and cognitive impairments (Sakharkar et al., 2016). Moreover, the observed reduction in CREB activity, a transcription factor crucial for learning and memory (Kida, 2023), provides further evidence of disrupted intracellular signaling pathways underlying cognitive deficits induced by adolescent alcohol exposure (Crews and Nixon, 2009). Collectively, these findings indicate that the prefrontal cortex is particularly sensitive to ethanol-induced epigenetic alterations, a vulnerability likely exacerbated by its ongoing maturation during adolescence (Crews et al., 2007; Barbier et al., 2015). Sustained transcriptional repression in this region may compromise executive and affective functions, thereby increasing the risk of anxiety and emotional disorders in adolescent drinkers (Longley et al., 2021). This mechanism could partly explain why ethanol exposure during adolescence predisposes individuals to heightened anxiety and escalated alcohol consumption at adulthood (Yang et al., 2013; Kyzar et al., 2019; Pandey et al., 2015; Pierrefiche, 2024).

In the hippocampus and striatum, binge-like alcohol exposure induced reductions in 5mC levels and altered histone deacetylase (HDACs) activity. Specifically, the hippocampus exhibited DNA hypomethylation associated with decreased DNA methyltransferase activity (*Dnmt1, Dnmt3a/b*), suggesting passive demethylation (Howell et al., 2001). Alcohol exposure also increased histone methyltransferases (*Ehmt1, Ezh2*), which may promote chromatin relaxation and impair memory functions (Zhang et al., 2023; Chen et al., 2011). Additionally, binge-like alcohol exposure stimulated histone acetyltransferases *Cbp, Kat7 gene expression* in the hippocampus, enhancing histone acetylation and DNA accessibility. Similar elevations in acetyltransferase levels have been reported in the amygdala following alcohol exposure linked to chromatin relaxation and reduced HDAC activity, which can contribute to anxiolytic effects (Pandey et al., 2017). Together, these findings suggest that adolescent alcohol exposure may compromise memory and emotional regulation through coordinated alterations in histone and DNA methylation patterns.

In the striatum, binge-like alcohol exposure promoted active demethylation via upregulation of *Tet1/2* enzymes, potentially reversing previous methylation changes and restoring gene expression (Moyon et al., 2021). The observed increases in *Tet1/2*, along with transient adjustments in histone methylation enzymes, such as elevated *Ezh2* and reduced *Ehmt1*, indicate adaptive epigenetic regulation aimed to counteract the effect of alcohol on reward processing and impulse control. These transcriptional changes in the striatum, a key region for reward and decision-making circuits (Cox and Witten, 2019) may heighten the risk of addiction. Persistent epigenetic modifications in this region could underlie behavioral adaptations that contribute to dependence and impaired impulse control (Wolstenholme et al., 2017).

In contrast, the cerebellum appears relatively resilient to acute alcohol exposure showing no global DNA methylation changes. Gene expression analyses revealed upregulation of DNA methyltransferases, i.e., *Dnmt1, Dnmt3a/b* and histone deacetylases *Hdac1/2*, promoting chromatin condensation and gene silencing (Turner, 2002; Hsieh and Gage, 2005). Concurrently, enzymes that oppose these silencing effects, including Ten-Eleven translocation enzymes (*Tet1/2*), histone methyltransferases (*Ehmt1, Ezh2*), and histone acetyltransferases (*Kat7, Cbp*), are also upregulated, suggesting a dynamic and biphasic regulation that maintains gene expression homeostasis in response to acute alcohol exposure (Abel and Zukin, 2008; Zhang et al., 2018; Wang et al., 2023). This dual regulation likely reflects a protective or compensatory mechanism, balancing gene silencing and activation to preserve cerebellar function and counteract alcohol-induced disruptions. Such resilience indicates that the cerebellum can maintain cellular homeostasis, potentially preventing maladaptive changes associated with alcohol exposure (Abel and Zukin, 2008; Turner, 2002; Pandey et al., 2017; Bohnsack and Pandey, 2021; Wang et al., 2023).

Behavioral studies indicate that P30 mice exposed to binge-like ethanol exhibited deficits in cognitive performance, reduced sensorimotor coordination and an anxiety-like behavior. Similar adolescent binge-like ethanol models have reported these behavior disruption without locomotor impairments (Fernandes et al., 2018; Reitz et al., 2021; De Oliveira et al., 2024). Thus, the reduced arm entries in our Ethanol-exposed mice likely reflect altered exploratory activity and anxiety rather than locomotor deficits. These functional impairments are likely associated with the epigenetic alterations in the prefrontal cortex, hippocampus and striatum, suggesting that modifications in DNA methylation and histone marks disrupt gene expression underlying learning, memory, and emotional regulation. In support of this notion, neuroanatomical studies have shown that early-life alcohol exposure induces structural changes in multiple brain regions, including the basal ganglia, thalamus, hippocampus, corpus callosum, and amygdala, many of which persist into adulthood and contribute to long-lasting behavioral deficits (Pandey et al., 2017; Kyzar et al., 2016). Repeated BD particularly impacts the amygdala, linking enduring anatomical and epigenetic alterations to learning impairments, deficits in adaptive functioning, and increased anxiety (Kyzar et al., 2016). Collectively, these findings indicate that alcohol-induced oxidative stress and epigenetic dysregulation during adolescence can directly contribute to persistent cognitive and behavioral impairments. Such developmental patterns suggest that BD-induced epigenetic changes may predispose individuals to long-term cognitive deficits, anxiety, and a higher risk of alcohol use disorder, AUD, in adulthood (Nestler, 2011; Longley et al., 2021; Pandey et al., 2015). Moreover, the sequential maturation of the brain from sensory to prefrontal regions may render adolescents particularly sensitive to alcohol’s deleterious effects on neural development (Spear, 2018; Deschamps et al., 2023; Brocato and Wolstenholme, 2021).

Acute binge-like ethanol exposure during adolescence induces rapid molecular disturbances within the first hours of exposure including alteration in protein networks, activation of oxidative and neuroimmune pathways, and early changes in synaptic structural markers such as synaptophysin and PSD-95 (Kyzar et al., 2016). These early events can affect synaptic stability, trigger glial reactivity, and initiate microglia-dependent synaptic remodeling, mechanisms known to rapidly influence neural function (Czerniawski and Guzowski, 2014; Pérez-Acuña et al., 2023). Such acute molecular responses may subsequently launch delayed cascades involving sustained shifts in microglial phenotype, altered neurotrophic support and epigenetic reprogramming of synaptic regulatory genes leading to the alteration of circuit connectivity and excitability over time (Hoogland et al., 2015; Wu et al., 2025). These processes provide a plausible mechanistic bridge leading to the behavioral impairments observed 96 h after exposure.

In our study, oxidative stress, epigenetic alterations, and behavioral changes occurred concurrently following a single ethanol exposure. Although a direct causal relationship between these molecular changes was not clearly established, oxidative stress has been shown to influence epigenetic regulation through redox-dependent modulation of DNMT and TET enzyme activity (Manev and Dzitoyeva, 2013; Dutta and Ruden, 2024). Moreover, adolescent alcohol exposure is known to induce long-lasting epigenetic alterations and behavioral distrubtion (Crews et al., 2016; Sakharkar et al., 2016). These findings highlight promising avenues for future work designed to determine whether oxidative stress directly contributes to the epigenetic remodeling and behavioral consequences associated with adolescent binge-like ethanol exposure. This study was limited to male adolescents to minimize variability from hormonal fluctuations and the known neuroprotective effects of estrogen which could influence the response of brain to alcohol (Amantea et al., 2005). Future studies including females will be conducted to assess whether sex-specific hormonal influences modulate the molecular, epigenetic, and behavioral responses to alcohol exposure.

This study highlights the significant and region-specific epigenetic impact of binge-like ethanol exposure on the adolescent brain. The pronounced vulnerability of the prefrontal cortex, together with the distinct epigenetic responses observed in the hippocampus and striatum, highlights the potential long-term consequences of adolescent binge-like alcohol exposure. In contrast, the relative cerebellum resilience to alcohol-induced methylation changes provides insight into region-specific protective mechanisms. These findings indicate that the developmental state of the adolescent brain shapes its response to alcohol, potentially contributing to the emergence of neuropsychiatric disorders and increased risk of addiction later in life. Investigating these epigenetic pathways further may inform the development of preventive strategies and therapeutic interventions for adolescent alcohol use disorder.

## Data Availability

The datasets presented in this study can be found in online repositories. The names of the repository/repositories and accession number(s) can be found in the article/Supplementary material.
