# Supplementary material for: Regional and temporal dynamics of DNA methylation and epigenetic gene regulation in response to binge-like alcohol exposure in the adolescent mouse brain
Source: Front Mol Neurosci. 2026 Jan 5;18:1716792. doi: 10.3389/fnmol.2025.1716792 (PMC12812982; doi:10.3389/fnmol.2025.1716792)
Supplement: Supplementary file 1 [file Data_Sheet_1.pdf]

				Prefrontal cortex		Hippocampus		Cerebellum		Striatum	
		Gene	Time (h)	Mean	Sem	Mean	Sem	Mean	Sem	Mean	Sem
key genes in DNA modification	DNA methyltransferases	Dnmt1	H4	+1,7	0	+0,7	0	-0,5	0	¬	
			H6	+0,8	0,1	+0,7	0,1	--	--	--	--
			H12	+.,3	0	+1	0,2	--	--	--	--
		Dnmt3a	H4	--	--	--	--	-0,8	0	--	--
			H6	--	--	+0,7	0,1	-0,6	0	--	--
			H12	+0,4	0	--	--	-0,5	0,1	--	--
		Dnmt3b	H4	--	--	--	--	--	--	--	--
			H6	+0,5	0,1	--	--			--	--
			H12	--	--	+0,7	0,1	-0,3	0	--	--
	Ten-eleven translocation	Tet1	H4	-0.3	0,1	+0,9	0,3	--	--	--	--
			H6	-0.6	0,1	--	--	--	--	--	--
			H12	-0.6	0	--	--	--	--	+1	0,2
		Tet2	H4	--	--	--	--	--	--	+1,4	0,4
			H6	-0.7	0	--	--	--	--	+0,8	0,1
			H12	-0.7	0	--	--	--	--		
key genes in histone modification	Histone methyltransferase	EZH2	H4	--	--	--	--	+0,8	0,2	+1,5	0,4
			H6	--	--	--	--	--	--	+0,9	0,1
			H12	--	--	+1,1	0,1	--	--		
		EHMT1	H4	--	--	--	--	+0,7	0,2	-0,4	0,1
			H6	--	--	--	--	+0,6	0,1		
			H12	--	--	--	--				
	Histone acetyltransferases	KAT7	H4	--	--	--	--	+1,2	0	+0,5	0,2
			H6	+0.6	0	--	--	+0,6	0,1		
			H12	-0.6	0,1	+0,4	0,1	--	--		
		CBP	H4					--	--	+0,8	0,2
			H6	-0.5	0	+1,1	0,3	--	--	--	--
			H12	--	--	--	--	+0,8	0,2	--	--
	Histone deacetylases	HDAC1	H4	+1.2	0,3	--	--	--	--	--	--
			H6	--	--	+0,5	0,1	--	--	-0,4	0
			H12	--	--	--	--	--	--	-0,4	0,1
		HDAC2	H4	--	--	+1,2	0,3	--	--	--	--
			H6	+0,7	0,2	+1,3	0,4	-0,4	0,1	--	--
			H12	+2,2	0,4	--	--	--	--	--	--
Behavioral disorder		Elevated Plus Maze				Reduced exploration		Bradykinesia			
		T-maze		Working memory impairment		Reduced spontaneous alternation				Habit-like behavior (no alternation)	
						[illegible]					
